# Facilitating Nitrite-Derived S-Nitrosothiol Formation in the Upper Gastrointestinal Tract in the Therapy of Cardiovascular Diseases

**DOI:** 10.3390/antiox13060691

**Published:** 2024-06-04

**Authors:** Mila Silva-Cunha, Riccardo Lacchini, Jose E. Tanus-Santos

**Affiliations:** 1 Department of Pharmacology, Ribeirao Preto Medical School, University of Sao Paulo, Ribeirao Preto 14049-900, Brazil; milacunha@usp.br; 2 Department of Psychiatric Nursing and Human Sciences, Ribeirao Preto College of Nursing, University of Sao Paulo, Ribeirao Preto 14040-902, Brazil; rlacchini@eerp.usp.br

**Keywords:** cardiovascular diseases, nitric oxide, nitrosation, nitrosothiols

## Abstract

Cardiovascular diseases (CVDs) are often associated with impaired nitric oxide (NO) bioavailability, a critical pathophysiological alteration in CVDs and an important target for therapeutic interventions. Recent studies have revealed the potential of inorganic nitrite and nitrate as sources of NO, offering promising alternatives for managing various cardiovascular conditions. It is now becoming clear that taking advantage of enzymatic pathways involved in nitrite reduction to NO is very relevant in new therapeutics. However, recent studies have shown that nitrite may be bioactivated in the acidic gastric environment, where nitrite generates NO and a variety of S-nitrosating compounds that result in increased circulating S-nitrosothiol concentrations and S-nitrosation of tissue pharmacological targets. Moreover, transnitrosation reactions may further nitrosate other targets, resulting in improved cardiovascular function in patients with CVDs. In this review, we comprehensively address the mechanisms and relevant effects of nitrate and nitrite-stimulated gastric S-nitrosothiol formation that may promote S-nitrosation of pharmacological targets in various CVDs. Recently identified interfering factors that may inhibit these mechanisms and prevent the beneficial responses to nitrate and nitrite therapy were also taken into consideration.

## 1. Introduction

Cardiovascular diseases (CVDs) are a group of disorders associated with high mortality and morbidity. According to the World Health Organization (WHO), 17.9 million people died from CVDs in 2019, that is 32% of all global deaths [1]. Given the critical role that nitric oxide (NO) plays in cardiovascular homeostasis, the modulation of the NO signaling pathway has been proved as a major strategy that needs to be explored in the therapy of diseases involving the cardiovascular system [2]. NO is the primary vasorelaxant stimulus of endogenous origin, and its action opposes various contractile stimuli. Its tonic production is continuous and at a low level, maintaining normotension in homeostatic conditions, during which the activity of nitric oxide synthase (NOS) is reduced [3].

NO synthesis is catalyzed by at least three synthases, neuronal (NOS1), inducible (NOS2), and endothelial (NOS3). These enzymes convert arginine into citrulline, producing NO in the process. The NOS1 and NOS3 isoforms are constitutively expressed, while the isoform NOS2 is induced particularly by immune and inflammatory stimuli [4]. Another way to produce NO is through activation of the nitrate enterosalivary cycle, which enables the formation of NO from nitrate and nitrite ingested in the diet. The NO-related species formed in the stomach through this pathway can participate in reactions involving the nitrosation of thiol-containing compounds and increase the formation of S-nitrosothiols (RSNO) [5,6].

Thus, apart from the direct generation of NO, several NO-related species contribute to its biological action. These species include nitrite (NO_2_^−^), nitrate (NO_3_^−^), and S-nitrosothiols [7]. Endogenous nitrite and nitrate generated as oxidation products of L-arginine-derived NOS activity can be enzymatically reduced back to NO under specific conditions, providing an alternative source of NO, especially during hypoxic conditions [8]. The formation of NO and S-nitrosothiols from both nitrate and nitrite is particularly relevant because S-nitrosothiols act as reservoirs of NO, releasing it in a controlled and localized manner when needed [6]. Understanding the mechanisms of NO-related species generation and their functions is essential for deciphering the intricate network of NO-related signaling in biological systems [9].

NO stimulates soluble guanylate cyclase (sGC) enzyme activity in vascular smooth muscle cells (VSMCs), leading to the formation of cGMP, which activates cGMP-dependent protein kinase (PKG), an enzyme that phosphorylates several cellular substrates involved in cell signaling. This mechanism induces relaxation of vascular smooth muscle and decreases peripheral vascular resistance, thus contributing to a reduction in blood pressure. Moreover, NO also exerts antihypertrophic activity in the long term, playing a critical role in preventing cardiac hypertrophy and vascular remodeling [10,11,12]. In addition to directly activating GCs, NO can participate in several chemical reactions that alter protein functions. One such post-translational modification is S-nitrosation, which involves the covalent attachment of NO to specific cysteine residues in proteins, forming S-nitrosoproteins (SNO-proteins) like S-nitrosoglutathione (GSNO). Proteins involved in platelet functions and mitochondrial proteins can also undergo nitrosation [13,14].

This review aims at providing current evidence concerning the gastric formation of S-nitrosothiols, with a focus on exploring the effects of NO-mediated signaling in the treatment of CVDs. To achieve this goal, a search was conducted in the PubMed and ScienceDirect databases for original articles using specific terms: ‘gastric nitrosothiols formation’, ‘cardiovascular effects of S-nitrosothiols’, ‘enterosalivary cycle of nitrate’, ‘exogenous nitrite or nitrate supplementation’, and ‘deficiency in nitric oxide bioavailability’. The selection criteria for articles included in this study were full publication in English and preferably published within the last 10 years to ensure the most current information on the subject.

## 2. Deficiency in NO Bioavailability and Activity in Cardiovascular Diseases

Research has demonstrated that NO plays a critical role in maintaining cardiovascular health. NO plays a pivotal role in vasodilation, anti-inflammatory responses, and inhibition of platelet aggregation. Deficiency in the availability of NO has been recognized as a significant contributor to the development and progression of various CVDs [15,16]. The NO produced by NOS3 participates in the regulation of physiological and pathological vascular responses [17]. Regulation of vascular tone is mediated by the interaction of NO with sGC in VSMCs. When sGC is activated in these cells, it leads to increased levels of cGMP [18,19]. cGMP-induced VSMC relaxation is mainly mediated by PKG activation, which can phosphorylate and inactivate Ca^2+^ channels and the Ins(1,4,5)P3-receptor-associated cGKI substrate (IRAG) located at the endoplasmic reticulum membrane [20].

These actions lead to a reduction in intracellular Ca^2+^ concentration, essential for vascular smooth muscle contraction. The influx of Ca^2+^ and release of Ca^2+^ reserves from the endoplasmic reticulum, mediated by IRAG, favor the initiation of contraction by activating the Ca^2+^/calmodulin-dependent myosin light-chain kinase (MLCK), which phosphorylates myosin light chain (MLC), and consequently, activates myosin ATPase. This enzyme uses adenosine triphosphate (ATP) as a source of energy to move along actin filaments [21]. This contractile pathway is inhibited by the increase in cGMP levels mediated by NO [22]. Thus, NO produced by the endothelium acts on VSMCs, promoting their relaxation, which reduces resistance to blood flow, resulting mainly in reduced cardiac workload and lower blood pressure [23]. Additionally, NO produced by the endothelium can activate platelet sGC and promote the phosphorylation of vasodilator-stimulated phosphoprotein, which is activated and inhibits platelet adhesion [24,25].

Studies have revealed that impaired function of these cells contributes to increased blood pressure by reducing NO production, increasing oxidative stress and inflammation. These factors collectively contribute to impaired vasodilation, increased vascular resistance, and ultimately the development and progression of hypertension and its deleterious consequences [17,26].

**Figure 1:**
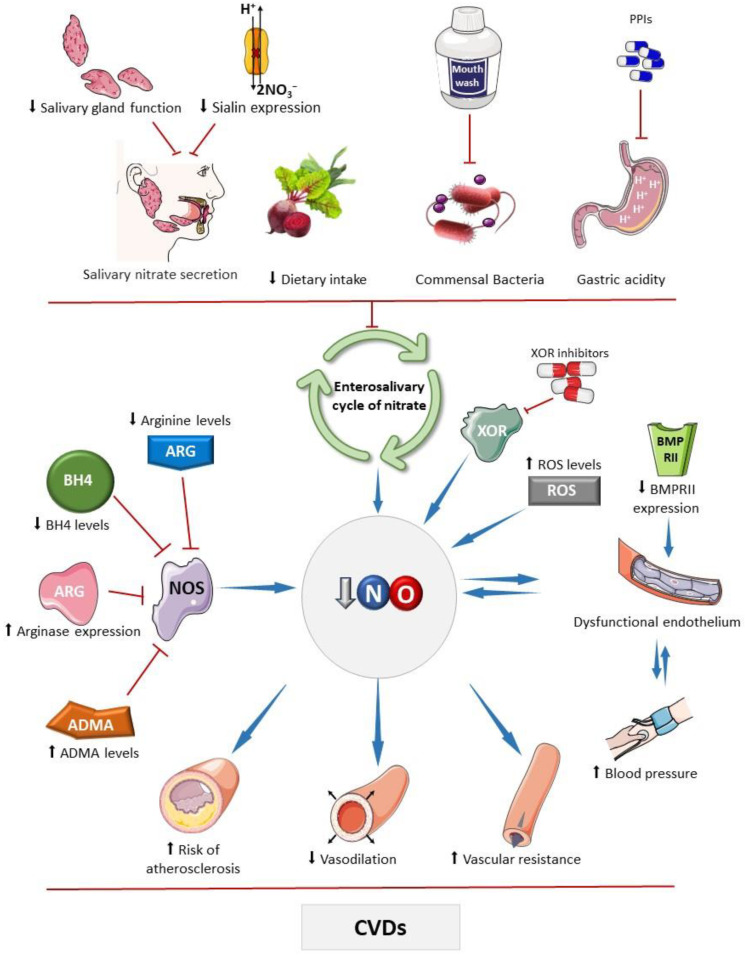
Main factors that affect the bioavailability of nitric oxide. The bioavailability of nitric oxide (NO) can be influenced by various factors that impact the activity of the standard NO synthesis pathway, carried out by nitric oxide synthases (NOSs). These factors include reduced levels of its substrate, arginine, or its cofactor tetrahydrobiopterin (BH4), and increased expression of arginase and levels of asymmetric dimethylarginine (ADMA), which competes with arginine for interaction with the enzyme [31,32,33,34,35]. Drugs that inhibit enzymes with nitrite reductase activity, such as xanthine oxidoreductase (XOR), can affect NO, as seen with febuxostat and allopurinol, which may inhibit nitrite-derived NO formation [36]. Elevated oxidative stress can deplete NO due to diffusion-limited NO reaction with superoxide producing peroxynitrite, thereby decreasing bioavailable NO levels. Chronic high blood pressure can lead to endothelial damage, and subsequently reduce NO production [37]. Similarly, other factors that diminish NO bioavailability may compromise physiological vasodilation and contribute to hypertension development. Bone morphogenetic protein receptor II (BMPRII) plays a role in endothelial cell survival and proliferation; reduced expression of this receptor has been linked to the development of endothelial dysfunction [31,32,33,34,35]. Factors that impair the enterosalivary nitrate cycle also decrease NO bioavailability, including ablation or dysfunction of salivary glands, which secrete nitrate into the oral cavity, and genetic disorders affecting the nitrate cotransporter in these glands (sialin) [38]. Reduced dietary intake of nitrate or nitrite, caused by decreased consumption of vegetables rich in these compounds, frequent use of mouthwash that reduces the commensal microbial population with nitrate reductase activity, and the use of proton pump inhibitors (PPIs), which attenuate non-enzymatic nitrite reduction to NO in the stomach, may reduce the NO bioavailability [39,40]. These mechanisms may compromise NO-mediated vasodilatory effects, increasing peripheral vascular resistance, and promote pro-atherosclerotic mechanisms that are associated with cardiovascular diseases. Parts of the figure were drawn using pictures from Servier Medical Art. Servier Medical Art by Servier is licensed under a Creative Commons Attribution 4.0 unported license.

Senescence is another important factor that contributes to the development of endothelial dysfunction. With advanced age, NOS3 activity is reduced in comparison to that in younger individuals, with the loss of efficient NO production [27]. These are some of the reasons why senescence and hypertension are cardiovascular risk factors. In addition, the upregulation of the enzyme arginase, which catalyzes arginine degradation, the substrate of the NO synthases, was observed in CVDs [28]. Supplementation with arginine or inhibition of arginase favors the formation of NO and its bioavailability, as demonstrated by previous studies (Figure 1) [29,30].

Recently published research evaluated NOS3 expression in cells subjected to unidirectional and multidirectional shear stress. The results show a positive correlation between NOS3 expression and shear stress, emphasizing the endothelium’s ability to protect itself in response to the tension generated by the friction force of blood flow. Therefore, a dysfunctional endothelium is more prone to damage, and consequently, the formation of atheroma plaques [41]. The dysfunctional endothelium promotes the accumulation of cholesterol and inflammatory cells within arterial walls, triggering the development of atherosclerotic plaques [42]. These plaques narrow the arteries, restricting blood flow and potentially lead to serious cardiovascular events including heart attacks, ischemic heart disease, and stroke [43]. Addressing endothelial dysfunction is crucial to mitigate these outcomes, highlighting its significance in preventing and managing issues related to arterial stiffness and plaque buildup [44].

Deficient NO bioavailability and activity compromise cGMP formation and its actions as a cellular messenger. Drugs that activate sGC serve as allosteric agonists, enhancing sGC sensitivity to endogenous NO and consequently boosting cGMP production. These drugs play an antihypertensive role and are also useful for treating cardiovascular diseases characterized by a reduction in NO plasma levels. The increase in sensitivity to NO, even at low concentrations, is pivotal to its effects [45]. Recently, a study [46] has demonstrated that treatment with praliciguat, a stimulator of sGC, reduced systolic blood pressure by up to 10 mmHg in individuals with hypertension and diabetes after 14 days of therapy. These results corroborate the hypothesis that increased cGMP availability attenuates hypertension associated with endothelial dysfunction observed in type 2 diabetes.

The multicenter randomized VICTORIA study included over 5000 patients with heart failure with reduced ejection fraction treated with vericiguat, a novel stimulator of sGC. The results revealed more remarkable survival in patients who received the drug than those who received a placebo [47]. In heart failure, there may be reduced blood supply to tissues, including the kidneys, due to decreased cardiac output. This leads to an increased activity of the angiotensin-converting enzyme (ACE) in kidney tubular epithelial cells, increasing the formation of angiotensin II (AngII) and the degradation of bradykinin, which can generate generalized vasoconstriction and contribute to the development of endothelial dysfunction and the formation of reactive oxygen species (ROS) [48,49]. Both conditions are associated with reduction in NO bioavailability, resulting in decreased generation of cGMP.

While drugs that inhibit the degradation of cGMP are not usually used in patients with hypertension, they can be utilized in pulmonary arterial hypertension, such as the phosphodiesterase inhibitors sildenafil and tadalafil [50]. An impaired NO pathway is frequently associated with the development of this disease through different mechanisms, including pulmonary endothelial dysfunction, reduced NOS3 activity, decreased expression of the bone morphogenetic protein receptor II (BMPRII), increased symmetric and asymmetric dimethylarginine (ADMA) levels or arginase I, and tetrahydrobiopterin (BH4; a cofactor involved in NOS activity) or arginine deficiency [31,32,33,34,35].

High levels of ADMA, an endogenous inhibitor of NOS, reduce the formation of NO. Under hypoxic conditions, there is an increase in the activity of dimethylarginine dimethylaminohydrolase (DDAH), the enzyme responsible for metabolizing ADMA, thereby increasing the availability of NO [51,52]. Moreover, elevated levels of ROS decrease NO levels. Superoxide (a major ROS) reacts with NO and generates peroxynitrite (ONOO^−^), an important oxidizing agent which causes critical damage to cells. Therefore, increased oxidative stress increases NO consumption and promotes peroxynitrite formation [37]. BMPRII, in turn, is involved in regulating angiogenesis, and proliferation and survival of endothelial cells. The reduction of its expression is associated with endothelial dysfunction and is observed in idiopathic pulmonary arterial hypertension (Figure 1) [31].

In addition to impaired activation of the classical NO-cGMP pathway, the reduced bioavailability of NO also affects nitrosation reactions, which still are not yet fully understood. However, reduced serum S-nitrosothiol levels were observed in patients with acute coronary syndrome compared to the control group [53]. S-nitrosothiols have a much longer lifetime than NO, making them an excellent means of storage and preventing inactivation by metalloproteins and free radicals.

Previous studies have explored mechanisms of nitrosation of receptors to explain the cardiovascular effects of S-nitrosothiols. β-adrenergic receptors (β-ARs) are G-protein-coupled receptors (GPCRs) that control the mechanisms of cardiac chronotropism and inotropism. GPCR kinases (GRKs) curtail G-protein signaling and target receptors for internalization. In cells, S-nitrosothiols and NO synthases may nitrosate Cys340 residues of GRK2, reducing GRK2-mediated β-AR phosphorylation with subsequent recruitment of β-arrestin to the receptor. This would result in the attenuation of receptor desensitization and internalization. Therefore, it is becoming clear that promoting nitrosation mechanisms may be a molecular strategy to prevent a reduction in the density of these receptors [54,55]. A number of additional targets for S-nitrosation remain to be studied.

## 3. The Enterosalivary Cycle of Nitrate as an Alternative Route to Generate NO

**Figure 2:**
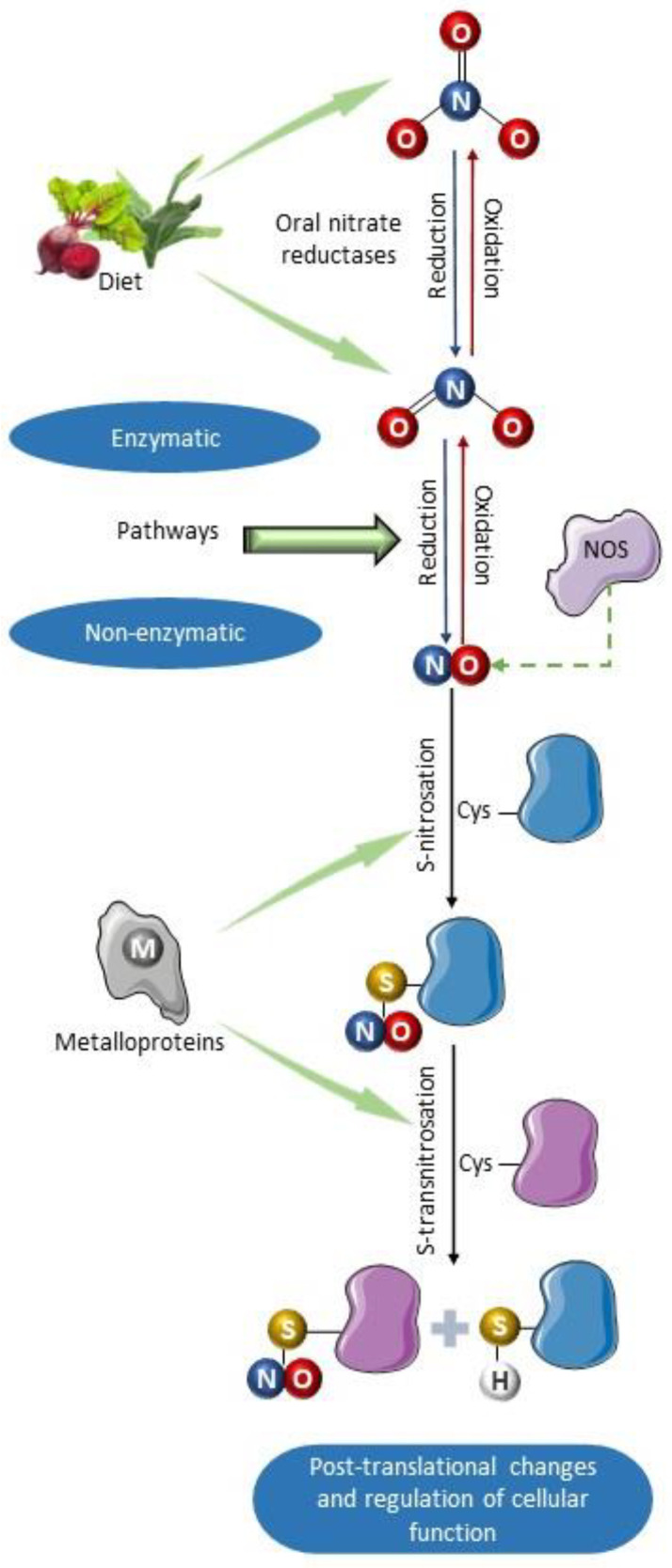
Schematic summary of the primary formation pathways of S-nitrosothiols. Nitric oxide can be synthesized by nitric oxide synthases and by non-enzymatic mechanisms involved in the enterosalivary cycle of nitrate [72]. It can also be produced as a result of enzymatic nitrite reductase activity of deoxyhemoglobin, endothelial alpha globin, xanthine oxidoreductase (XOR), aldehyde oxidase (AO), sulfite oxidase (SO), and moonlighting enzyme mitochondrial amidoxime-reducing component (mARC) [61,62,63,64,65,66,67]. Once formed, NO can react with various protein substrates containing residues of the amino acid cysteine, which can be nitrosated producing S-nitrosoproteins (nitrosothiols). Subsequently, S-transnitrosation reaction can transfer the nitro group from one thiol group to another, thus nitrosating other proteins. Such nitrosation reactions can be catalyzed by metalloproteins such as cytochrome C, ceruloplasmin, and iron–sulfur centers [73]. This molecular modification produced by nitrosation alters the function of proteins, modulating their activity. Parts of the figure were drawn using pictures from Servier Medical Art. Servier Medical Art by Servier is licensed under a Creative Commons Attribution 4.0 unported license.

The NO synthesized by NOS can undergo oxidation to nitrite and nitrate in the bloodstream. The multicopper oxidase ceruloplasmin may mediate this oxidation reaction, which converts NO to the nitrosonium cation (NO^+^), which is susceptible to a hydration reaction forming nitrite [56]. NO is also oxidized to nitrite through a reaction with oxyhemoglobin, which can also oxidize nitrite to nitrate [57], and therefore, nitrate is the most abundant product of NO oxidation found in plasma, usually found at much higher concentrations than nitrite. However, the reverse pathway, involving reductive reactions converting both nitrate and nitrite to NO, can also occur [58,59]. In the systemic circulation and tissue organs, many proteins are capable of catalyzing the NO formation from nitrite as a result of their nitrite reductase activity [60]. These proteins include deoxyhemoglobin, myoglobin, neuroglobin, endothelial alpha globin, xanthine oxidoreductase (XOR), aldehyde oxidase (AO), sulfite oxidase (SO), NOS3, and mitochondrial enzymes, such as the moonlighting enzyme mitochondrial amidoxime-reducing component (mARC) [61,62,63,64,65,66,67]. Similar to XOR, SO, and AO, mARC contains a metallic center with molybdenum (Mo), serving as a catalyst for redox reactions. The reduction of nitrite to NO is facilitated by Mo through electron transfer, resulting in the oxidation of Mo (IV) to Mo (V) [68,69]. Some of these enzymes are upregulated under hypoxic conditions, when there is an increased demand for NO formation and improved blood flow (Figure 2) [70,71].

In addition to nitrite reduction by proteins with nitrite reductase activity, the enterosalivary cycle of nitrate also supports NO generation. In this cycle, circulating nitrate reaches the oral cavity via the salivary glands. Certain bacteria in the oral microbiome produce enzymes that can act as nitrate reductases, converting nitrate to nitrite. The nitrite is then further reduced to NO in the acidic environment of the stomach. This pathway is strongly activated by nitrate and nitrite derived from the diet [39]. Additionally, the stimulated NO formation in the stomach can nitrosate various proteins and result in increased circulating levels of S-nitrosothiols, which may function as a strategy to prolong the half-life of NO and guarantee its availability during hypoxic conditions [74]. Any agents that interfere in the functionality of the enterosalivary cycle of nitrate decrease S-nitrosothiol formation and impair the antihypertensive effects of nitrate and nitrite [5].

Intriguingly, the enterosalivary cycle of nitrate not only acts as a crucial source of NO but also underscores the complex relationship between diet, the oral microbiome, and cardiovascular health. Recent research has emphasized the potential impact of dietary choices and oral hygiene practices on this pathway. For instance, diets rich in nitrate-containing foods such as leafy greens and beets can enhance nitrate availability for this cycle, presenting a potential natural dietary method to increase NO production [72]. Conversely, disturbances in the oral microbiome or conditions that affect saliva production might diminish the effectiveness of this pathway. Furthermore, manipulating this cycle through targeted interventions, such as probiotics or dietary adjustments, shows promise in preventing and managing cardiovascular disease [75]. Continued exploration of the enterosalivary cycle of nitrate and its role in NO biology could offer valuable insights into innovative strategies for promoting cardiovascular health and addressing related conditions [72,76,77].

## 4. Dietary Nitrite and Nitrate Enhance Gastric Nitrosothiol Formation

S-nitrosothiols are molecules resulting from the reaction between NO^+^ and a thiolate anion (RS^−^). S-nitrosothiols with low molecular weight are formed from compounds such as cysteine and glutathione, while those with high molecular weight include S-nitrosoalbumin and S-nitrosohemoglobin [73]. Our studies have shown that oral supplementation with inorganic nitrate or nitrite in hypertensive rats increases the gastric formation of S-nitrosothiols associated with antihypertensive effects [5,9,78]. The oral administration of beetroot juice can also increase plasmatic levels of S-nitrosothiols due to its high nitrate and nitrite concentration [79]. Similarly, other dietary sources of nitrate and nitrite include lettuce, spinach, rocket, tatsoi, garland chrysanthemum, laver, and shiitake mushrooms, which are commonly found in Mediterranean and Japanese diets [80].

The nitrite reaching the stomach is converted to NO, participating in nitrosation or transnitrosation reactions. Nitrosation requires either NO or thiol oxidation, which can be facilitated by metalloproteins including ceruloplasmin [81], cytochrome C [82], and other proteins with iron–sulfur centers [83] (Figure 2). In the stomach, this reaction can occur following the intake of nitrate or nitrite [84]:

NO_2_^−^ + H^+^ ↔ HNO_2_ (1)

GSH + HNO_2_ ↔ GSH + NO + OH^−^ ↔ GSNO + H_2_O (2)

Interestingly, the effects of nitrite treatment on the formation of nitrosothiols were potentiated by low doses of ascorbate and attenuated by high doses [85]. This observation might be linked to changes in the redox environment of the stomach, which affect nitrosation reactions. Indeed, the hypotensive responses of nitrate and nitrite were associated with increased plasma levels of S-nitrosothiols and vascular S-nitrosation of pharmacological targets involved in blood pressure regulation [86,87].

The mechanisms involved in gastric S-nitrosothiol formation are well-established. Previous studies investigating S-nitrosothiol levels in gastric aspirates from healthy humans treated with potassium nitrate or sodium nitrite showed significant increases in the gastric levels of S-nitrosothiols compared to basal levels [84,88]. In this context, a new mechanism has been proposed for some antiplatelet drugs of the thienopyridine class, such as clopidogrel and prasugrel, which involves the formation of S-nitrosothiols from nitrite in acidic conditions [89]:

Thienopyridine-SH + H^+^ (Stomach) + NO_2_^−^ (Saliva and stomach) ↔ Thienopyridine-SNO + H_2_O (3)

This mechanism is supported by in vivo evidence, demonstrating an acute increase in plasma levels of S-nitrosothiols after treatment with prasugrel in patients with known coronary artery disease. Therefore, thienopyridines may act as inhibitors of ADP P2Y12 receptors, serving as antiplatelet agents, and, when nitrosated, as NO donors. This study indicates intriguing new mechanisms of action including known cardioprotective effects of NO. New studies can better clarify these new mechanisms of action [90].

Once formed, S-nitrosothiols can react with RSH, transferring NO from one thiol compound to another:

RSNO + R’SH ↔ RSH + R’SNO(4)

This reaction, known as transnitrosation, is involved in regulating proteins, such as the reaction between SNO-protein and either glutathione (GSH) to form GSNO or thioredoxin (Trx) to form SNO-Trx [73]. GSNO is an essential source of NO due to the high concentration of GSH in cells, its longer half-life, and its tendency to undergo transnitrosation [91,92]. SNO-Trx may transnitrosate the Cys163 residue of caspase-3, thereby modulating its pro-apoptotic action [93]. 

Through transnitrosation, new S-nitrosothiols and thiols can be generated and activate biological functions [94]. For example, S-nitrosothiols play a role in gastric mucosal defense, potentially preserving the integrity of the stomach lining and providing protection against damage induced by ROS or other harmful agents [95,96].

Currently, some techniques for S-nitrosoprotein detection allow the identification not only of abundant proteins such as GSNO, but also less abundant proteins present in different compartments, at nM concentrations [97]. Liquid chromatography coupled with mass spectrometry (LC-MS) is the most employed technique in nitrosoproteomic analysis, especially when associated with assays that stabilize and isolate such proteins, such as the biotin switch assay, resin-assisted capture, two-dimensional gel electrophoresis, and methods using anti-S-NO antibodies [98,99]. These methodologies contribute to elucidating physiopathological mechanisms involving the modulation of S-nitrosothiol formation, enabling the identification of targets for pharmacological interventions.

## 5. Exogenous Nitrite or Nitrate Supplementation as a Strategy to Activate the Enterosalivary Cycle of Nitrate and Supplement Endogenous NO Formation

Considering the impact of reduced NO bioavailability on the pathophysiology of CVDs, strategies aimed at compensating for this deficit may yield benefits for cardiovascular health. Some of these strategies focus on the NO canonical pathway, involving the modulation of NOS action and the inhibition of cGMP degradation [100,101]. However, the enterosalivary cycle of nitrate is known to serve as an additional source of NO, complementing the canonical pathway. This offers a promising approach to enhance NO bioavailability and potentially mitigate the pathophysiological mechanisms involved in CVDs [102,103].

Organic nitrates and nitrites are synthetic compounds created through reactions between an alcohol and an acid, such as nitric acid, resulting in a hydrocarbon with the nitrate or nitrite (R-ONO_2(3)_) [104]. These substances serve as NO donors; nitroglycerin, for example, is a vasodilator drug [105]. Nevertheless, the mechanism by which inorganic nitrate and nitrite function differ. Unlike direct NO donors, these compounds act as substrates for enzymatic or non-enzymatic conversion, leading to NO formation. This process can be beneficial as it supports a physiological pathway and helps to prevent potential collateral effects [106].

In this regard, a previous study compared the effects of organic and inorganic nitrates on aortic and carotid hemodynamics in heart failure [107]. The results indicated that nitroglycerin induced vasodilation in the carotid circulation, reducing blood pressure, but had inconsistent effects on central wave reflections. On the other hand, inorganic nitrate consistently reduces wave reflections without causing significant hypotension or dilatation in the cerebrovascular system, which is commonly associated with the adverse effect of headaches linked to nitroglycerin use. These findings align with previous studies demonstrating an improvement in exercise capacity in these patients following treatment with inorganic nitrate. Moreover, they highlight an advantage of inorganic nitrate over organic nitrate due to its fewer adverse effects [108].

That being said, a meta-analysis comprising human studies concluded that dietary nitrate supplementation has several positive effects, including reduced resting blood pressure, improved endothelial function, decreased arterial stiffness, and reduced platelet aggregation [109]. Moreover, beyond their vasodilatory effect, nitrate and nitrite also display antioxidant properties, aiding in shielding against oxidative stress and inflammation, both pivotal factors in the development and progression of CVDs [110,111,112].

It is worth noting that supplementing with either nitrate or nitrite is possible. The primary differences between the two forms lie in their oxidation states. Nitrate must be reduced to nitrite before reaching the stomach, whereas nitrite is rapidly converted to NO. Consequently, nitrate may exhibit greater oral bioavailability, while nitrite might demonstrate better acute effects [113,114,115]. Further studies are needed to clarify relevant differences between them. However, supplementation with inorganic nitrite has shown promise in improving endothelial function associated with aging. As age increases, there is a rise in oxidative stress and a decline in mitochondrial function, which is linked to the development of endothelial dysfunction and a decrease in the bioavailability of NO. Oral treatment with inorganic nitrite appears to attenuate these effects [116,117].

In addition to the therapeutic perspective, the supplementation or consumption of dietary nitrate has been shown to prevent CVDs. However, this preventive effect might be influenced or affected by various factors. For example, increased nitrate intake or consumption of leafy greens was associated with decreased risk of hypertension [118]. However, exposure to ambient particulate matter (PM) can attenuate this beneficial effect due to increase in oxidative stress caused by PM, which is associated with reduced NO bioavailability. These particulates include organic matter, heavy metals, and other toxic substances that potentially interfere with the mechanism of nitrate bioactivation, leading to increased NO bioavailability [119,120,121,122,123,124,125]. Next, we will discuss agents that may affect the enterosalivary cycle of nitrate, and consequently, the bioavailability and activity of NO.

## 6. Agents Impairing the Enterosalivary Cycle of Nitrate

Several agents have been identified that can disrupt the enterosalivary nitrate cycle, interfering with the conversion of nitrate to nitrite, and subsequently, affecting the generation of NO. Some conditions or primary agents known to impair the enterosalivary nitrate cycle include antimicrobials, impaired salivary gland secretion of nitrate, antiseptic mouthwashes, and increased gastric pH [126,127,128,129,130]. Reduced NO production resulting from impairment of the enterosalivary nitrate cycle can contribute to various conditions, including hypertension, endothelial dysfunction, and increased susceptibility to infections [131].

### 6.1. Impaired Salivary Gland Secretion of Nitrate

The salivary transport of nitrate is crucial for maintaining NO homeostasis as it represents a critical stage in the enterosalivary cycle of nitrate. Interestingly, nitrate concentrations are higher in saliva than in plasma [132], and this fact reflects the activity of sialin [38], which acts as an electrogenic cotransporter of nitrate (2NO_3_^−^/H^+^) in the plasma membrane of salivary gland acinar cells. Knockdown of sialin expression results in reduced nitrate transport, leading to decreased nitrate secretion in saliva, even following a nitrate-rich diet (Figure 1). Mutations in the sialin gene are linked to Salla disease and infantile sialic acid storage disorder, a severe autosomal recessive lysosomal storage disorder characterized by early physical and mental impairment [133,134,135]. However, there is currently a lack of studies investigating the potential impact of the nitrate–nitrite–NO pathway and its clinical consequences in individuals affected by these diseases.

The mechanism of nitrate transport in saliva is not fully understood, but it is recognized that there is competition with iodine, perchlorate, and thiocyanate for uptake and concentration in saliva [136]. This competition might be related to the function of the sialin cotransporter [135]. In addition, numerous factors, particularly individual variations, can influence salivary nitrate secretion. The parotid gland is the primary source of nitrate secretion in saliva, and experiments involving the removal of the parotid glands have shown a loss of active secretion of nitrate from blood into saliva [137]. The ablation or reduced function of this gland may be observed in patients undergoing treatment for oral or head and neck cancers, particularly those treated with radiotherapy [138,139]. In patients with Sjögren’s syndrome, an autoimmune disease primarily affecting the salivary and lacrimal glands, dysfunction or damage to these glands can lead to reduced saliva and tear production [140]. Damage to the function of the salivary glands can occur in patients treated with antineoplastic drugs, including cyclophosphamide [141] and 5-fluorouracil [36], or with antibiotics, such as metronidazole [142], or analgesic drugs, such as tramadol [143]. Other agents known to affect salivary glands include ethanol [78] and heavy metals [144,145]. It remains to be studied how many other agents may affect salivary glands and nitrate secretion, possibly interfering with the enterosalivary cycle of nitrate.

### 6.2. Impaired Oral Nitrate Reduction to Nitrite with the Use of Oral Mouthwash

After ingestion or secretion by the salivary glands, nitrate is reduced to nitrite by bacteria with nitrate reductase enzymes usually found in the oral microbiota [146,147]. Oral hygiene agents with antimicrobial properties can influence the bacterial population, potentially disrupting the nitrate conversion pathway. Mouthwashes containing chlorhexidine, renowned for their antibacterial effects, have been extensively studied regarding their impact on oral microbiota. Many studies consistently indicate a reduction in nitrite formation from nitrate when these agents are used, thus leading to a decreased NO production rate [148,149,150]. The impaired nitrate reduction to nitrite clearly impacts the beneficial effects of nitrate on the cardiovascular system, especially its antihypertensive action [30,151,152]. Maybe this interference in the responses to nitrate may be circumvented by administering oral nitrite, as previously demonstrated by our group [86].

In hospitalized patients, mouthwash use is frequently recommended to prevent respiratory and systemic complications, especially in intensive care units with high risk of nosocomial pneumonia [153]. However, some studies, including a meta-analysis, have linked the use of chlorhexidine-containing mouthwash with increased mortality in these patients [154,155,156]. While some studies linked higher concentrations of chlorhexidine in mouthwash solutions (around 2%) with oral mucosal ulceration and increased mortality [157,158], it is possible that the investigation of the potential correlation between mouthwash usage, its effects on the nitrate–nitrite–NO pathway, and increased mortality could elucidate a causal relationship. The suppression of oral nitrate reductase activity due to mouthwash usage affects the bioavailability of NO, which plays a crucial role in maintaining homeostasis, and thus, contributes to impairment of the recovery process in patients [159].

### 6.3. Impaired Nitrite Reduction to NO and Decreased Formation of Nitrosating Species in the Stomach by the Use of Drugs That Increase Gastric pH

Nitrite is reduced to NO through non-enzymatic mechanisms in the acidic environment of the stomach [160]. This process encompasses several pathways, including the acidic breakdown of nitrite to create nitrous acid (HNO_2_), which subsequently reacts further to produce NO and other nitrosating species including the nitrosonium ion (NO^+^), nitrogen dioxide (NO_2_), and dinitrogen trioxide (N_2_O_3_) [161]. However, drugs that increase the gastric pH, such as PPIs like omeprazole [40,162] or histamine H2-receptor antagonists (H2RAs) like ranitidine [163], have been associated with impaired nitrite reduction to NO and other nitrosating species within the stomach [5]. It is now becoming clear that drugs such as PPIs or H2RAs prevent oral nitrite-induced systemic consequences, particularly those mediated by NO signaling related to the generation of nitrosating species [164,165,166].

In fact, the gastric formation of NO and other chemical species was shown in a seminal study more than four decades ago [167]. The authors showed that treatment with omeprazole blunted 95% of gastric NO formation. In the acidic pH, nitrite is protonated and forms nitrous acid (HNO_2_), which can generate the nitryl ion (H_2_NO_2_^+^) in the presence of H^+^. This nitryl ion is in equilibrium with the formation of NO^+^ and water. The nitryl ion may react with nitrite to form N_2_O_3_, and its dissociation generates NO and NO_2_. The reaction is described as follows [168]:

NO_2_^−^ + H^+^ ⇌ HNO_2_ (5)

HNO_2_ + H^+^ ⇌ H_2_NO_2_^+^ ⇌ NO^+^ + H_2_O (6)

H_2_NO_2_^+^ + NO_2_^−^ ⇌ N_2_O_3_ + H_2_O (7)

N_2_O_3_ ⇌ NO + NO_2_ (8)

The consequences of indiscriminate use of PPIs or H2RAs on the cardiovascular system have been the focus of studies, which demonstrated reduced bioavailability of NO and RXNO (nitrosylated species) after treatment with both classes of drugs [164,169]. Clinically, chronic use of these drugs may be associated with endothelial dysfunction, hypertension, and the development of other CVDs [162,170]. Moreover, increased pH compromises the antihypertensive and cardioprotective effects of nitrite supplementation and reduces the formation of S-nitrosothiols, which drive the antihypertensive effects of nitrite (Figure 1) [5,160,171].

Another mechanism proposed to explain the effects of PPIs on the cardiovascular system involves the inhibition of the enzyme DDAH. This inhibition leads to the accumulation of ADMA, which is a classical NOS inhibitor [172,173]. However, there is some controversy regarding this issue because some authors concluded that PPIs are weak and reversible DDAH inhibitors in vitro at clinical concentrations [174]. Additionally, in vivo there were no significant associations between PPIs and ADMA. Further studies are necessary to clarify the mechanisms involved in these effects.

## 7. Pharmacological Strategies to Facilitate Gastric Nitrosothiol Formation

The S-nitrosation of proteins is a major mechanism by which NO regulates cellular function. However, mechanisms that explain a possible specificity of protein S-nitrosation are still unknown, even though it is clear that different thiol groups have variable susceptibilities to S-nitrosation, which is influenced by local chemical conditions [175]. In 2022, researchers from Qingdao University in China released a proteomic database with multiple post-translational modifications of cysteine named CysModDB^®^. This database contains 8237 proteins modified by S-nitrosation in humans [176]. This approach may prove to be very helpful in improving our understanding of the complex biological implications of such post-translational modifications. The gastric formation of S-nitrosothiols that takes place after nitrate or oral nitrite administration requires further investigation given its physiological and pharmacological relevance. Understanding the cardiovascular health benefits of NO and therapeutic strategies that increase S-nitrosothiol formation at the gastric level could be advantageous [177].

**Figure 3:**
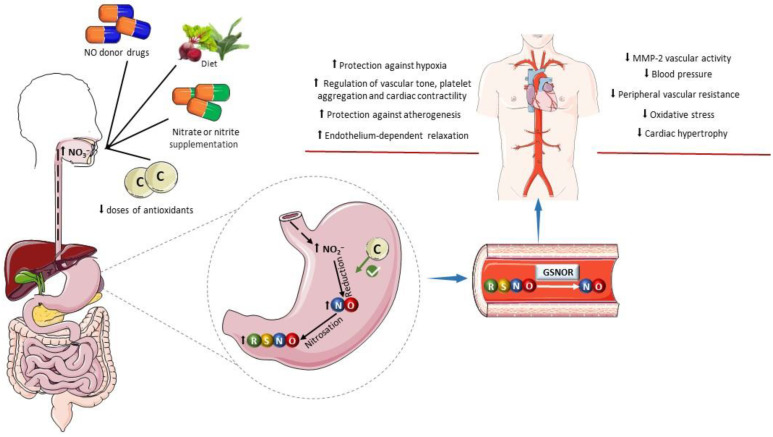
Influence of dietary factors on the formation of S-nitrosothiols. The oral administration of drugs acting as exogenous NO donors can increase the gastric formation of nitrosothiols (RSNOs) and promote nitrosation of a variety of pharmacological targets. Similarly, an increase in the intake of inorganic nitrate and nitrite in the diet or by oral supplementation enhances gastric NO formation and RSNO formation. Low doses of antioxidants can favor the reduction of nitrite to nitric oxide (NO) in the stomach. Examples include ascorbic acid, tempol, and retinol. In plasma and tissues, RSNOs may undergo denitrosylation catalyzed by S-nitrosoglutathione reductase (GSNOR) releasing NO or promote transnitrosylation of pharmacological targets. The cardiovascular benefits of this process include antioxidant effects, protection against hypoxia, improved regulation of the vascular tone and blood pressure, decreased platelet aggregation, increased cardiac contractility, protection against atherogenesis, decreased vascular activity of matrix metalloproteinase-2 (MMP-2), and attenuation of cardiovascular remodeling. Parts of the figure were drawn using pictures from Servier Medical Art. Servier Medical Art by Servier is licensed under a Creative Commons Attribution 4.0 unported license.

The use of exogenous NO donors represents a notable pharmacological strategy to increase NO levels in the gastric mucosa, thereby fostering the generation of nitrosothiols. When administered orally, compounds such as nitroglycerin, sodium nitroprusside, and other NO-releasing agents have been shown to enhance systemic S-nitrosothiol levels. This mechanism explains at least part of the effects of these drugs and potentially extends the duration of their effects. Similarly, as detailed above, supplementation with inorganic nitrate and oral nitrite promotes the formation and systemic effects of NO donors (Figure 3) [79].

Certain dietary compounds and nutritional interventions have been examined for their potential to positively impact gastric nitrosothiol formation. While many vegetables abundant in nitrate and nitrite contribute to activate the nitrate–nitrite–NO pathway and to the formation of S-nitrosothiols in the stomach, some of these vegetables are also rich in polyphenols, which may contribute to S-nitrosothiol formation, mainly due to their antioxidant and reductive actions. Supporting this notion, studies have investigated the effect of the combination of nitrite or nitrate with various antioxidants such as retinol, alpha-tocopherol, ascorbic acid, TEMPOL (4-hydroxy-2,2,6,6-tetramethylpiperidine-N-oxyl), and beta-carotene [78,84,178,179]. These studies have shown increased S-nitrosothiol formation compared to treatments involving only nitrite or nitrate (Figure 3). As an important example, the following reaction shows how ascorbate facilitates NO formation from nitrite, leading to the oxidation of ascorbate to dehydroascorbic acid (DHA) [180]:

AscH^−^ + 2NO_2_^−^ + 3H^+^ → DHA + 2NO^•^ + H_2_O (9)

The augmentation of the gastric formation of NO and other nitrosating species facilitates reactions with proteins and leads to the generation of S-nitrosothiols, probably facilitating the therapeutic effects of nitrite and nitrate. Interestingly, this action depends on the dose of ascorbate, as low doses of ascorbate in conjunction with oral nitrite treatment increase S-nitrosothiol formation and the antihypertensive responses to nitrite, whereas high doses of ascorbate exhibit the opposite effect [85]. This difference with respect to the dose of ascorbate is probably explained by the ability of ascorbate to decompose GSNO and release NO [181].

Additionally, sulfur-containing amino acids, particularly cysteine, play a significant role in augmenting S-nitrosothiol formation due to their thiol content [182]. In this context, the discussion about nitrite-induced generation of nitrosamines arises. The nitrosation of the amino group of amino acids generates nitrosamines, known for their carcinogenic potential, especially concerning the digestive system. In fact, long-term exposure to nitrate and nitrite, used as preservatives in processed meats by the food industry, has been associated with an increase in cancer cases [183]. However, oral treatment with sodium nitrite and nitrate demonstrates beneficial effects on the cardiovascular system and overall health [184], similar to the Mediterranean diet, which is rich in these substances and is considered healthy, contributing to increased life expectancy in that region [185,186].

The formation of nitrosamines has been investigated during the storage of foods or even during their processing, which means individuals might ingest the actual nitrosamine and not just its precursors [187]. This could explain results presented by the French NutriNet-Santé study, a cohort that included 101,056 adult participants. It demonstrated a significant association between the intake of nitrate and nitrite from processed foods and cancer diagnosis. However, it did not associate intake from natural sources with the same outcome [188]. It is important to note that some studies indicate an inhibitory effect on the formation of nitrosamines from nitrate and nitrite when accompanied by antioxidants such as alpha-tocopherol [189], selenium [190], and ascorbic acid [191,192], similar to what naturally occurs in vegetables [193]. Indeed, vitamin C is included in the composition of excipients of drugs, especially in formulations that have the potential for nitrosamine formation [194,195]. The same principle applies to meats, where legislation in many countries recommends the addition of vitamin C to products preserved with nitrite and nitrate [196,197]. Moreover, NO^+^ is a ‘soft’ electrophile that preferably reacts with sulfur compared to nitrogen [198,199]. Therefore, the reaction in the stomach from nitrite favors S-nitrosation over N-nitrosation. This mechanism is enhanced by antioxidants, generating S-nitrosothiols, whose cardioprotective effects have been recognized in recent decades [78,200].

## 8. Pharmacological Cardiovascular Effects of Increased S-Nitrosothiol Concentrations

Increases in S-nitrosothiol concentrations promote enhanced bioavailability of NO because circulating S-nitrosothiols can be degraded to release NO in the blood or into tissues. This cleavage can be catalyzed by enzymes such as GSNO reductase (GSNOR) (Figure 3). GSNOR is the primary cellular denitrosylase, catalyzing the reduction of GSNO in a two-step reaction that requires NADH as a cofactor. This process releases glutathione disulfide (GSSG) and the NO moiety as ammonia (NH_3_) or hydroxylamine (NH_2_OH) [201,202,203].

GSNOR GSNO + NADH → GSNHOH + NAD^+^ (10)

GSNHOH + GSH → NH_2_OH (or NH_3_) + GSSG (11)

The cleavage of S-nitrosothiols also releases NO through chemical reactions with other compounds such as hydropersulfides (RSSH) [204] and metals [205,206]. In addition to being unstable in the presence of metals or when exposed to light [207], these compounds can also undergo decomposition through photolysis, a homolytic process that forms NO and the thiol radical [208]. On the other hand, metals promote heterolytic cleavage, which does not produce thiol radicals and may involve biologically active reducing agents such as transition metal ions, such as copper, or other agents including ascorbate, superoxide, and thiols [209].

The increased bioavailability of NO resulting from higher concentrations of S-nitrosothiols usually improves functional aspects of the cardiovascular system. For example, a variety of different S-nitrosothiols were shown to promote reduced vasoconstriction in response to an α1-adrenergic agonist, such as phenylephrine [177], thus suggesting that S-nitrosothiols may restore endothelium-dependent relaxation that is impaired by endothelial dysfunction. These findings align with those reported in other studies showing a correlation between increased plasma concentrations of S-nitrosothiols and blood pressure reduction, especially following oral treatment with inorganic nitrite or nitrate [5,210,211].

Nitrosothiols have been shown to exert antioxidant effects [212], which may affect blood pressure regulation. Indeed, hypertension is frequently linked to exacerbated oxidative stress, which can result in endothelial dysfunction and hindered NO signaling [213]. As antioxidants, nitrosothiols can scavenge ROS and aid in restoring the bioavailability of NO. By preventing peroxynitrite formation, they facilitate vasodilation, thereby reducing blood pressure [214]. Moreover, nitrosothiols might influence other physiological pathways in blood pressure regulation, such as the renin–angiotensin–aldosterone system and sympathetic nervous system activity. However, the exact mechanisms by which they interact with these systems require further investigation and clarification [215,216,217]. It has recently been shown that oral nitrite-induced increases in circulating nitrosothiol concentrations result in increased S-nitrosylation of vascular protein kinase C, which is a critical intracellular signaling mediator of AngII vasoconstriction [161]. Moreover, the same treatment apparently prevents alfa-1 adrenergic receptor-mediated vasoconstriction by enhancing S-nitrosylation of calcium/calmodulin-dependent protein kinase II, another important mediator of vasoconstriction. These studies point to S-nitrosylation of pharmacological targets as another major mechanism involved in the effects of oral nitrite.

Nitrosothiols function as a NO reservoir that can release NO as necessary. With respect to heart function, nitrosothiols may affect cardiac contractility and relaxation, thus influencing overall cardiac performance. One of the mechanisms by which nitrosothiols enhance heart function involves their ability to regulate calcium handling in cardiac muscle cells. This is because calcium ions play a pivotal role in controlling the contraction and relaxation of the heart muscle and nitrosothiols modulate calcium sensitivity by altering the activity of proteins responsible for calcium handling, such as troponin C. These modifications ultimately affect the contractile characteristics of the heart [218,219].

Again, by diminishing oxidative stress, nitrosothiols contribute to sustaining the appropriate redox balance within the heart. This action aids in preserving cardiac function and acts as a protective mechanism against oxidative damage [220]. Studies conducted in animal models, as well as clinical research, have shown the advantageous effects of nitrosothiols in diverse cardiovascular conditions, including heart failure [221], ischemic heart disease [222], and hypertension [210]. Approaches directed at augmenting nitrosothiol bioavailability or focusing on nitrosothiols’ specific mechanisms of action show promise as potential therapeutic interventions to enhance heart function and mitigate cardiovascular diseases (CVDs). Interestingly, mitochondria-targeted S-nitrosothiol inhibits mitochondrial complex I in ischemic tissue reperfusion, preventing the formation of free radicals, thus protecting the heart against post-myocardial infarction heart failure [221]. It is now becoming clear that S-nitrosation mechanisms leading to cardioprotection involve the S-nitrosation or transnitrosation of mitochondrial proteins, as previously detailed [223,224,225]. However, while previous studies have shown a decreased infarcted area after ischemia and improved heart functionality [223,224,225], it remains to be proved whether oral nitrite-induced nitrosation results in clinical protection.

S-nitrosothiols may mediate protection against structural alterations associated with CVDs, such as vascular remodeling observed in hypertension [226]. Vascular remodeling encompasses structural and functional alterations in blood vessels, involving changes in their size, shape, and composition [227]. Nitrosothiols influence vascular remodeling through their ability to modulate vascular tone and regulate blood flow. They serve as NO donors, causing vasodilation and helping to regulate blood pressure and appropriate blood flow to tissues and organs [228]. Additionally, NO derived from nitrosothiols can inhibit the proliferation of VSMCs, which is crucial to prevent excessive thickening of blood vessel walls, a characteristic feature of vascular remodeling. The proposed mechanism to explain this effect involves disrupting the cell cycle of VSMCs by decreasing the levels of the ubiquitin-conjugating enzyme, which is crucial for mitosis. This effect has been observed both in vitro and in vivo [229,230,231].

As antioxidants, nitrosothiols also counteract vascular remodeling caused by oxidative stress by preventing the oxidation of crucial cellular components, thereby preserving vascular integrity [232]. Vascular matrix metalloproteinase-2 (MMP-2) activity, which is activated by oxidative stress, was inhibited by oral nitrite treatment, thus preventing hypertension-induced vascular remodeling [226]. Therefore, it is possible that increased S-nitrosothiol formation after oral nitrite treatment increases circulating levels of these mediators [5], which in turn nitrosate vascular MMP-2, decreasing its activity. Another possibility is that antioxidant effects of nitrosothiols prevent MMP-2 activation by hypertension-induced oxidative stress.

Furthermore, nitrosothiols have been implicated in regulating inflammatory responses within the vasculature. They can modulate the activity of various inflammatory mediators, cytokines, and adhesion molecules, thereby mitigating inflammation-induced vascular remodeling processes [233]. Inflammatory conditions increase the expression of matrix metalloproteinases (MMPs) [234], which participate in the remodeling of various tissues. Regarding the cardiovascular system, MMP-2 and MMP-9 are among the most important MMPs showing increased expression associated with hypertrophy of vascular smooth muscle and myocardium [235]. Treatment with S-nitrosothiols inhibits the translocation of NF-kB, and therefore, reduces the expression of pro-inflammatory cytokines that induce MMP-9 activity [236]. However, MMP nitrosation requires more detailed studies, as S-nitrosothiols were shown to activate (not to inhibit) MMP-9 [237]. There surely must be concentration-related issues with this mechanism.

Cardiac remodeling involves imbalanced MMP activity, which can also be mitigated or counteracted by NO. Cardiac hypertrophy may result from the chronic strain on the heart in hypertension. The proliferation of cardiac cells is coordinated by the protein kinase B and mammalian target of rapamycin (Akt/mTOR) signaling pathway, whose activation is favored by ROS [238].

In this respect, treatment with oral nitrite exerted antioxidant effects and reversed hypertension-induced cardiac hypertrophy in two-kidney one-clip (2K1C) rats [12]. The results demonstrate that nitrite reduces ROS levels in cardiac tissue and inhibits the mTOR pathway, consequently attenuating cardiac hypertrophy. It remains to be determined whether this effect is really mediated by S-nitrosothiols.

Previous studies have demonstrated the effect of NO on components of atherosclerotic plaque, highlighting a protective role against atherogenesis. Among these components are macrophages [239], smooth muscle cells [240], and endothelial cells [241]. The vasodilatory effect of NO alone is a protective mechanism against this disease due to its ability to reduce shear stress and potential endothelial injury, which can trigger plaque formation [242]. Additionally, the anti-inflammatory and antioxidant properties of S-nitrosothiols are also protective factors that can inhibit the activation of inflammatory pathways and reduce the adhesion of immune cells to the endothelium [243], as well as diminish oxidative stress, and subsequently, the oxidation of low-density lipoproteins (LDLs), thereby preventing plaque formation [244].

Curiously, Ref. [245] showed that GSNO or inhibition of GSNOR (which results in increased nitrosothiol concentrations) reduced the proliferation of T cells and the production of inflammatory cytokines observed in atherosclerosis. This effect involves S-transnitrosation of the Cys224 residue of Akt, preventing phosphorylation at Ser473, and thereby hindering the activation of the signaling cascade initiated by Akt. This pathway regulates the proliferation of T cells, their metabolism, survival, and production of pro-inflammatory cytokines. The reduced number of T cells and macrophages infiltrating atherosclerotic plaques is another important consequence of promoting S-nitrosation.

## 9. Conclusions

The evidence presented in this review suggests that pharmacological manipulation of gastric nitrosothiol synthesis is a promising therapeutic strategy for preventing and treating CVDs. The intricate pathways involving enzymatic processes, gastric transformations, and the enterosalivary cycle of nitrate underscore a complex interplay influencing cardiovascular health. The significant potential of S-nitrosothiols as mediators and their diverse roles in modulating cardiovascular function and structure highlight their impact on cardiovascular physiology and therapeutics. Optimizing the pathways that govern NO production from nitrite and nitrate, including S-nitrosothiol formation, is a pivotal step in enhancing therapeutic interventions. This review provided an updated overview of recent evidence supporting the role played by gastric S-nitrosothiol formation in the enterosalivary cycle of nitrate, which shapes the therapeutic effects of oral nitrite and nitrate. Future studies and clinical trials investigating these pathways hold promise in revolutionizing cardiovascular care and advancing personalized treatment modalities for individuals affected by CVDs.
